# Nutraceutical Value of Black Cherry *Prunus serotina* Ehrh. Fruits: Antioxidant and Antihypertensive Properties

**DOI:** 10.3390/molecules181214597

**Published:** 2013-11-25

**Authors:** Francisco J. Luna-Vázquez, César Ibarra-Alvarado, Alejandra Rojas-Molina, Juana I. Rojas-Molina, Elhadi M. Yahia, Dulce M. Rivera-Pastrana, Adriana Rojas-Molina, Miguel Ángel Zavala-Sánchez

**Affiliations:** 1 Doctorado en Ciencias Biológicas y de la Salud, Universidad Autónoma Metropolitana, Unidad Xochimilco, México D.F. 04960, Mexico; E-Mail: fjlunavz@yahoo.com.mx; 2 Laboratorio de Investigación Química y Farmacológica de Productos Naturales, Facultad de Química, Universidad Autónoma de Querétaro, Querétaro Qro.76010, Mexico; E-Mails: rojasa@uaq.mx (A.R.-M.); jirojasmolina@gmail.com (J.I.R.-M.); 3 Laboratorio de Fitoquímicos y Nutrición, Facultad de Ciencias Naturales, Universidad Autónoma de Querétaro, Querétaro Qro.76230, Mexico; E-Mails: yahia@uaq.mx (E.M.Y.); dulceriverap@hotmail.com (D.M.R.-P.); 4 División de Estudios de Posgrado, Facultad de Ingeniería, Universidad Autónoma de Querétaro, Querétaro Qro. 76010, Mexico; E-Mail: arojas@uaq.mx; 5 Departamento de Sistemas Biológicos. Universidad Autónoma Metropolitana, Unidad Xochimilco, México D.F. A.P. 23-181, Mexico; E-Mail: mzavala@correo.xoc.uam.mx

**Keywords:** *Prunus serotina*, black cherry, polyphenols, antioxidant capacity, vasorelaxant effect, antihypertensive effect

## Abstract

In Mexico black cherry (*Prunus serotina* Ehrh.) fruits are consumed fresh, dried or prepared in jam. Considering the evidence that has linked intake of fruits and vegetables rich in polyphenols to cardiovascular risk reduction, the aim of this study was to characterize the phenolic profile of black cherry fruits and to determine their antioxidant, vasorelaxant and antihypertensive effects. The proximate composition and mineral contents of these fruits were also assessed. Black cherry fruits possess a high content of phenolic compounds and display a significant antioxidant capacity. High-performance liquid chromatography/mass spectrometric analysis indicated that hyperoside, anthocyanins and chlorogenic acid were the main phenolic compounds found in these fruits. The black cherry aqueous extract elicited a concentration-dependent relaxation of aortic rings and induced a significant reduction on systolic blood pressure in L-NAME induced hypertensive rats after four weeks of treatment. Proximate analysis showed that black cherry fruits have high sugar, protein, and potassium contents. The results derived from this study indicate that black cherry fruits contain phenolic compounds which elicit significant antioxidant and antihypertensive effects. These findings suggest that these fruits might be considered as functional foods useful for the prevention and treatment of cardiovascular diseases.

## 1. Introduction

Cardiovascular diseases (CVD) are increasing throughout the world and are responsible for 34% of annual deaths that occur in low and middle-income countries [1]. Hypertension and other factors associated with modern lifestyle markedly increase the risk of CVD [2,3]. Despite the existence of several pharmacological approaches, the control of these pathologies is low [4]. Therefore, new strategies should be targeted at modifying lifestyle and nutritional habits to reduce the prevalence of CVD. A substantial body of evidence have linked intake of fruits and vegetables rich in polyphenolic compounds to cardiovascular risk reduction [5]. This beneficial effect is greatly attributed to the high antioxidant properties of polyphenols [6,7]. Although the most commonly used assays for determining antioxidant capacity very often give inconsistent results [8,9], several studies in animal models and in human subjects have actually confirmed that phenols are bioavailable and exert a protective role against oxidative stress and free radical damages [10,11]. Moreover, it has been demonstrated that these secondary metabolites have vasodilator [12] and antihypertensive properties [13,14], which involve other mechanisms apart from their antioxidant activity [15]. In this context, Mexico, with its floristic and cultural richness, provides a valuable source of plants with nourishing and medicinal properties. One of the plants whose use by the Mexican people dates from before the Spanish conquest is *Prunus serotina* Ehrh. (Rosaceae), a 60 to 90 foot-tall native North American tree with edible fruits [16]. This species, commonly called “capulín”, American black cherry, Virginian prune and bird cherry, is widely distributed in Western and Central Mexico and is used in the Mexican Folk Medicine for the treatment of several illnesses. Its leaves are used for treating hypertension, bronchitis, cough, and gastrointestinal disorders, such as diarrhea. Its fruits are used for the treatment of diarrhea and cough [17,18]. Besides their use in traditional medicine, the fruits of this plant are also part of Mexican diet, and they are consumed fresh, dried or prepared in jam [17].

Black cherry fruits have been the subject of few chemical studies. Anthocyanidins have been detected in the peel of fruit that was obtained from trees from Central Mexico [19]. Recently, as a part of a study directed towards the screening of fruits consumed in Ecuador, Vasco *et al*. [20,21] evaluated the antioxidant capacity and identified by liquid chromatography coupled to mass spectrometry (LC-MS) the phenolic compounds present in this species.

Our research group has found that the lyophilized aqueous and methanolic extracts obtained from black cherry leaves promoted vascular smooth muscle relaxation of the rat aorta [22]. Later, we identified three known natural products: hyperoside, prunine, and ursolic acid from the methanolic extract of the black cherry leaves. These compounds elicited a concentration-dependent relaxation of vascular smooth muscle. These results suggested that the health benefits of black cherry leaves for the treatment of hypertension are attributed, at least partly, to the presence of these secondary metabolites [23].

Taking into account this background, we hypothesized that black cherry fruits possess a high nutritional value and have polyphenolic and vasorelaxant compounds that could lower blood pressure. Therefore, the aim of this study was to characterize the phenolic profile of black cherry fruits obtained from Central Mexico and to determine their antioxidant activity as well as to investigate their vasorelaxant and antihypertensive effects. Moreover, the proximate composition and mineral contents of these fruits were determined in order to assess their nutritional value.

## 2. Results and Discussion

Results of proximate analysis of black cherry, plum (*Prunus domestica* L.), and grape (*Vitis vinifera* L.) are presented in Table 1. The latter species were selected for comparative purposes since they are widely consumed fruits, rich in phenolics and with significant antioxidant capacity [6]. In comparison with plums and grapes, black cherry fruit contains high levels of proteins (four times higher) and it has higher levels of carbohydrates than plum. Thus, this fruit is a good source of nutrients. Protein content in *P. serotina* fruit is higher than that reported in the literature for plum (0.9% ± 0.03%), apricot (*Prunus armeniaca* L.) (1.4% ± 0.33%), peach (*Prunus persica* L. Batsch) (0.9% ± 0.01%), and grapes (0.72% ± 0.03%) [24]. Fiber and protein contents were higher than those reported by Morton [25] for black cherry fruit collected in Guatemala and Ecuador. The contents of K, Ca, Mg, and P in black cherry fruit were significantly higher than those in plum and grape (Table 2, *p* < 0.05). It means that black cherry fruit represents a complementary source of these minerals for Central Mexican diet.

**Table 1:** Proximate composition of black cherry fruit, plum, and grape *.

Component	Black Cherry	Plum	Grape
Moisture	81.18 ± 0.87 ^a^	87.29 ± 1.12 ^b^	81.83 ± 0.72 ^a^
Protein	2.10 ± 0.01 ^a^	0.49 ± 0.04 ^b^	0.46 ± 0.01 ^b^
Fat	0.05 ± 0.01 ^a^	0.04 ± 0.01 ^a^	0.03 ± 0.01 ^a^
Fiber	3.58 ± 0.03 ^a^	3.57 ± 0.03 ^a^	3.21 ± 0.45 ^a^
Ash	0.86 ± 0.11 ^a^	0.37 ± 0.03 ^b^	0.49 ± 0.07 ^b^
Carbohydrate ^+^	12.23 ± 0.79 ^a^	8.28 ± 1.02 ^b^	13.96 ± 0.33 ^a^

* crude content g/100 g; mean ± standard deviation (*n* = 3). ^a^ and ^b^: values in the same row followed by the same letter are not significantly different (*p* > 0.05); ^+^ Carbohydrate content was determined by the difference method.

Results of total phenolic content, total flavonoids, and antioxidant capacity (DPPH and FRAP) are shown in Table 3. The amount of total phenolics and total flavonoids in black cherry peel were larger than those in black cherry flesh. These results are in agreement with data that point out that peel has a higher phenolic content than flesh in different fruits from genus *Prunus* [26,27], and other fruits such as apples and grapes [28,29]. The phenolic and flavonoid contents in black cherry fruit were the highest ones compared with those values obtained from plums and grapes.

**Table 2:** Mineral composition of black cherry fruit, plum, and grape *.

Mineral	Black Cherry	Plum	Grape
Sodium	22.40 ± 1.60 ^a^	15.2 ± 2.40 ^a^	12.50 ± 0.49 ^a^
Potassium	184.30 ± 3.50 ^a^	81.30 ± 2.00 ^b^	96.60 ± 3.74 ^b^
Calcium	12.90 ± 1.90 ^a^	4.20 ± 0.15 ^b^	4.41 ± 0.21 ^b^
Magnesium	21.20 ± 0.20 ^a^	10.40 ± 1.20 ^b^	5.30 ± 0.20 ^c^
Phosphorous	28.10 ± 0.40 ^a^	13.50 ± 0.50 ^b^	1.39 ± 0.40 ^b^

* mg/100 g of fresh fruit; mean ± standard deviation (*n* = 5). ^a^ and ^b^: values in the same row followed by the same letter are not significantly different (*p* > 0.05).

**Table 3:** Total Phenolics, Flavonoids, and Antioxidant Capacity (DPPH and FRAP) in Black Cherry Fruit, Plum, and Grape ^a^.

Fruit/Part of Fruit	Total Phenolics (mg of GAE/100 g of FW)	Flavonoids (mg of CE/100 g of FW)	DPPH ^b^ (µmol TE/100 g of FW)	FRAP ^b^ (µmol TE/100 g of FW)
**black cherry**	362.2 ± 11.6	201.8 ± 12.1	2056.7 ± 108.0	1455.2 ± 92.5
flesh	325.1 ± 12.0	146.3 ± 8.0	1764.6 ± 170.4	1100.7 ± 35.4
peel	564.9 ± 10.9	259.5 ± 14.5	2681.6 ± 180.0	1991.4 ± 40.1
**plum**	218.0 ± 10.5	180.2 ± 8.3	1137.8 ± 40.1	737.2 ± 68.0
**grape**	160.9 ± 12.1	121.2 ± 4.9	1148.6 ± 102.1	593.8 ± 46.9

^a^ Data are presented as mean ± standard deviation of nine replications; GAE, gallic acid equivalent; CE, catechin equivalent; TE, Trolox equivalent; FW, fresh weight; ^b^ Pearson’s correlation coefficient was used to calculated the correlation between phenolic content and antioxidant capacity measurement: *r* = 0.875 for DPPH and *r* = 0.959 for FRAP.

Whole black cherry DPPH and FRAP values were higher than those obtained from plums and grapes. According to the literature, plums and grapes are fruits with high antioxidant capacity [6,30]; therefore, these results suggest that black cherry fruit has a similar or better antioxidant properties than those fruits. However, considering that evaluation of antioxidant capacity cannot be accurately determined by any single method [8,9], it is highly recommended to apply different methodological approaches to fully characterize the antioxidant activity of *P. serotina* fruit. Moreover, the black cherry phenolic content correlates with the antioxidant capacity of this fruit (*r* = 0.875 for DPPH and *r* = 0.959 for FRAP). Therefore, the presence of phenolic compounds largely would account for the higher antioxidant capacity of black cherry peel than that one of black cherry flesh (Table 3). These results suggest that black cherry fruits are a rich source of natural antioxidants. Additionally, phenolic compounds could also exert health-promoting properties through others mechanisms besides their antioxidant activity.

**Figure 1:**
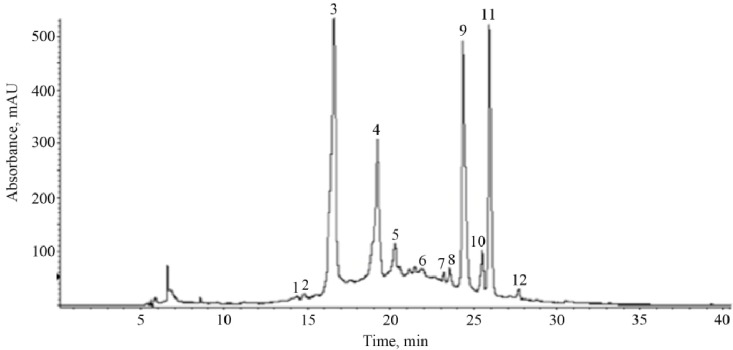
HPLC profile of phenolic compounds in black cherry peel. Peak numbers correspond to those in Table 4.

**Figure 2:**
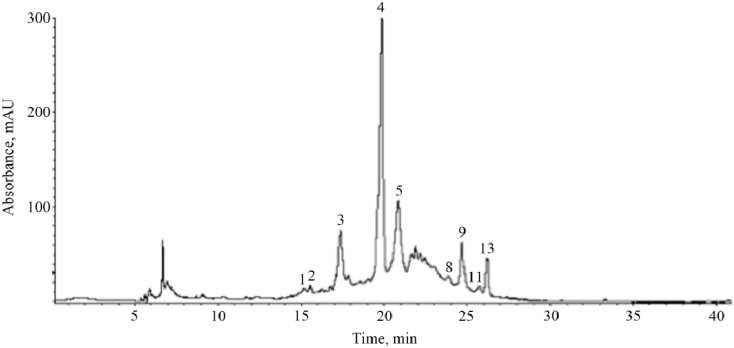
HPLC profile of phenolic compounds in black cherry flesh. Peak numbers correspond to those in Table 5.

**Figure 3:**
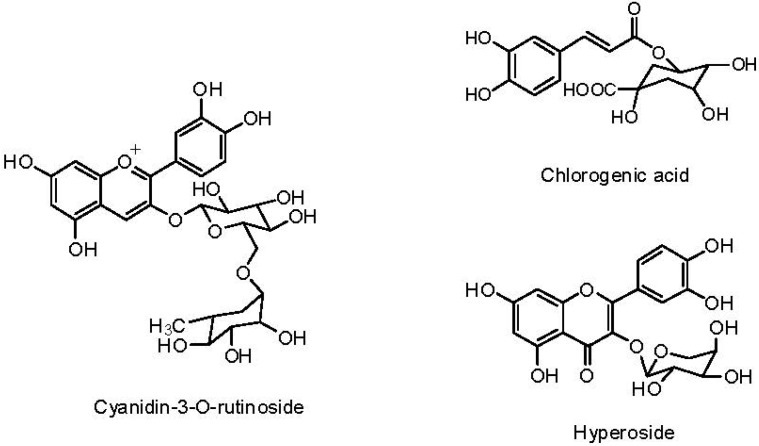
Chemical structures of major phenolic compounds.

Phenolic composition of fruits strongly depends on ripening, genetic, and environmental factors [27,31,32]. In the case of cherry fruits, it has been demonstrated that phenolic compounds contributing to their antioxidant capacity differ significantly across species and variety within species [32,33,34]. In this study, twelve phenolic compounds were identified in black cherry peel by HPLC-ESI-MS (Figure 1 and Table 4). Peak assignment of phenolic compounds in the chromatograms was based on the comparison of their UV-vis absorption spectra to those of the authentic samples and by their mass spectrometry fragmentation patterns obtained in negative ionization mode. According to HPLC-MS data analysis, cyanidin-3-O-rutinoside (peak 3), chlorogenic acid (peak 4), hyperoside (peak 9) and quercetin pentoside (peak 11) were the most abundant phenolic compounds in black cherry peel. The main phenolic compounds identified in black cherry flesh (Figure 2, Table 5) were cyanidin-3-O-rutinoside (peak 3), chlorogenic acid (peak 4), procyanidin B (peaks 5 and 8), hyperoside (peak 9) and quercetin malonilglucoside (peak 13). The presence of anthocyanins in the black cherry flesh could be due to the maturation stage of this fruit at the time of analysis. The major compounds identified in black cherry peel and flesh are shown in Figure 3.

**Table 4:** Retention times, UV-Vis, and mass spectral data of phenolic compounds in crude extract of black cherry peel.

Peak ^a^	t_R_ (min)	λ_max_ (nm)	Fragmentation Pattern [M-H]^−^	Compound
1	14.3	272	315 (169, 125)	gallic acid hexoside
2	14.8	292	329 (167, 152, 123, 108)	vanillic acid hexoside
3	16.6	284, 518	593 (465, 447, 285)	cyanidin-3-O-rutinoside
4	19.2	300, 328	353 (191)	chlorogenic acid
5	20.3	280	577 (425, 289)	procyanidin B (dimer)
6	22.3	296, 324	387 (179, 161, 135)	Caffeoyl hexose-deoxyhexoside
7	23.2	255, 354	609 (353, 301)	rutin
8	23.5	282	577 (425, 289)	procyanidin B (dimer)
9	24.3	256, 356	463 (301)	hyperoside
10	25.5	266, 348	447 (285)	kaempferol hexoside
11	25.9	256, 356	433 (301)	quercetin pentoside
12	27.7	264, 348	417 (285)	kaempferol pentoside

^a^ peak numbers correspond to Figure 1.

The content of quercetin glycosides was estimated as follows: hyperoside, 5.2 ± 0.4 mg/g dry weight and quercetin pentoside, 3.6 ± 0.4 mg/g dry weight in peel; hyperoside, 0.9 ± 0.2 mg/g dry weight and quercetin malonilglycoside, 0.3 ± 0.1 mg/g dry weight in flesh. Contents of the chlorogenic acid were estimated to be 2.9 ± 0.4 mg/g dry weight and 1.9 ± 0.3 mg/g dry weight in peel and flesh, respectively.

**Table 5:** Retention times, UV-Vis, and mass spectral data of phenolic compounds in crude extract of black cherry flesh.

Peak ^a^	t_R_ (min)	λ_max_ (nm)	Fragmentation Pattern [M-H]^−^	Compound
1	15.1	272	315 (169, 125)	gallic acid hexoside
2	15.5	292	329 (167, 152, 123, 108)	vanillic acid hexoside
3	17.3	284, 518	593 (465, 447, 285)	cyanidin-3-O-rutinoside
4	19.8	300, 328	353 (191)	chlorogenic acid
5	20.8	280	577 (425, 289)	procyanidin B (dimer)
8	23.9	282	577 (425, 289)	procyanidin B (dimer)
9	24.6	256, 356	463 (301)	hyperoside
11	25.7	256, 356	433 (301)	quercetin pentoside
13	26.2	256, 356	549 (505, 301)	quercetin malonilglucoside

^a^ peak numbers correspond to Figure 2.

These phenolic profiles were similar to those obtained by Vasco *et al*. [21] for this species from Ecuador. In both studies, chlorogenic acid was the main hydroxycinnamic acid in flesh and peel, and the presence of procyanidins type B, quercetin and kaempferol glycosides was detected as well. However, these authors found (+)-catechin, (−)-epicatechin, and proanthocyanidins as major compounds.

**Figure 4:**
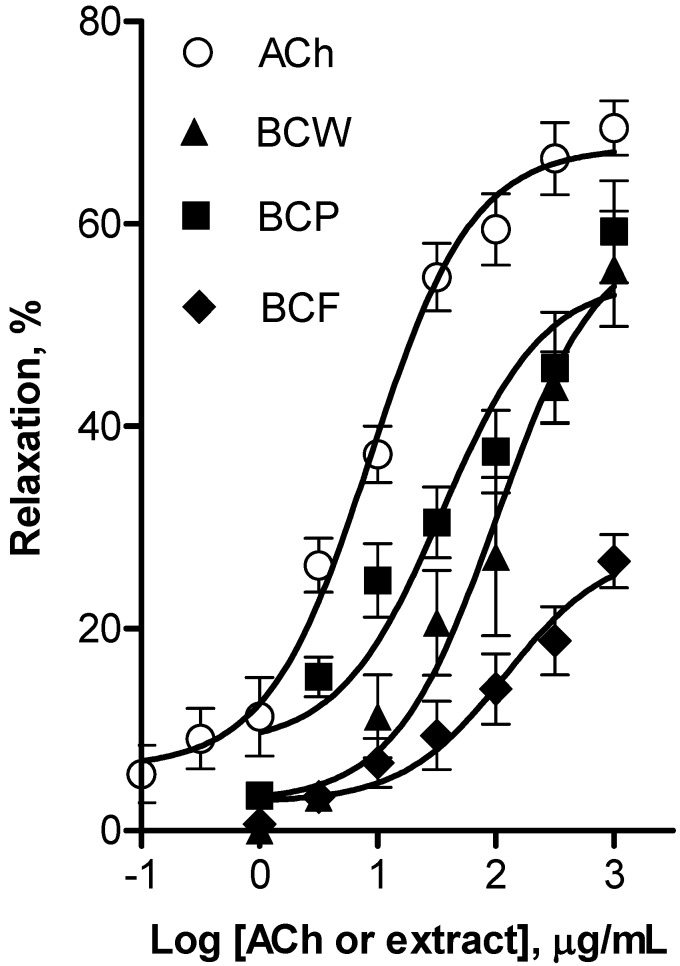
Concentration-response curves for the vascular relaxant effect of whole (BCW), peel (BCP) and flesh (BCF) black cherry and acetylcholine as positive control (ACh). These curves are presented as percentage of relaxation of rat aortic rings pre-contracted by l-phenylephrine (1 µM) as a function of extract concentration. All extracts elicited vasodilatation in a dose-dependent manner. Each individual value is a mean ± SEM (*n* = 6).

Considering that quercetin and cyanidin have high antioxidant activity, our results are consistent with the antioxidant capacity reported in this study. Moreover, these compounds have been related with other protective properties such as vasodilatory and antihypertensive effects besides their antioxidant capacity [12,35]. The results of the pharmacological evaluation employing the rat aorta model indicated that the lyophilized extracts obtained from whole fruit, peel, and flesh of black cherry elicited a concentration-dependent relaxation of aortic rings (Figure 4). The whole fruit (E_max_ = 59.0% ± 5.9%; EC_50_ = 101.8 ± 7.5 µg/mL) and peel (E_max_ = 54.5% ± 4.0%; EC_50_ = 34.9 ± 3.4 µg/mL) extracts of black cherry presented similar maximum vasorelaxant response, which were higher than that elicited by flesh extract (E_max_ = 27.9% ± 3.6%; EC_50_ = 120.0 ± 5.7 µg/mL). Whole fruit and peel extracts were approximately four fold less potent than ACh (EC_50_ = 8.6 ± 1.4 µg/mL). Nevertheless, both extracts elicited a maximum vasodilator effect similar to that of the positive control (E_max_ = 69.5% ± 5.7%). These results show for the first time that black cherry aqueous whole fruit and peel extracts elicited a significant smooth muscle relaxation on rat aorta.

According to the results obtained by HPLC-MS analysis, we consider that the vasodilator effect showed by aqueous peel extract is related, at least in part, to the presence of quercetin glycosides as these compounds have been shown to elicit a vasodilator effect on rat arteries [36,37]. Moreover, several studies have reported vasodilator effects elicited by phenolic compounds such as anthocyanins or proanthocyanidins [12,14]. Therefore, the vasodilator effect elicited by aqueous peel extract could be a synergistic response elicited by the phenolic compounds present in this extract.

**Figure 5:**
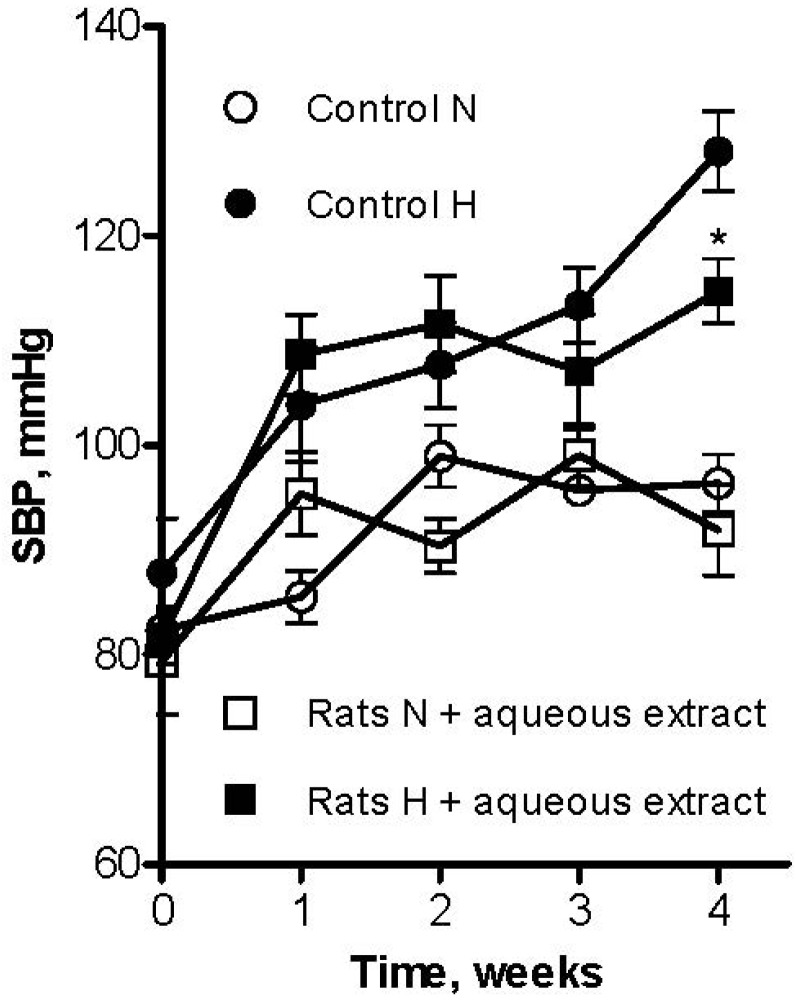
Effect of long-term oral administration of aqueous extract of black cherry fruits (300 mg/Kg/day) on systolic blood pressure (SBP) in L-NAME (30 mg/Kg/day) induced hypertensive rats (H), and normotensive rats (N). Control indicates rats receiving only tap water. Values are mean ± SEM (*n* = 6 rats for each group). * *p* ˂ 0.05 *vs*. control.

The lyophilized aqueous extract of black cherry fruits was orally administered to L-NAME-induced hypertensive and normotensive rats at a dose of 300 mg/kg body weight/day, which falls within the range of doses most frequently used to test anti-hypertensive effects of plant extracts in rats (100–700 mg/kg body weight/day) [38,39,40]. Mean value of systolic blood pressure (SBP) in control rats was 96 ± 3 mmHg (*n* = 6). As shown in Figure 5, treatment by L-NAME for four weeks increased SBP to 128 ± 4 mmHg (*n* = 6). However, the aqueous extract induced a significant reduction on systolic blood pressure (114 ± 3 mmHg) in L-NAME induced hypertensive rats. Furthermore, normotensive rats that received the aqueous extract had no significant change on SBP (92 ± 4 mmHg, *p* ˂ 0.05).

The dose of the lyophilized BCAE used in rats is equivalent to a dose in humans of 49 mg/kg body weight/day, according to an interspecies scaling from rats to humans based on the body surface area [41]. Considering that the lyophilized BCAE contains 35 ± 4 mg/g phenolic compounds, 49 mg of this extract contains 17 mg phenolic constituents, which corresponds to a daily consumption of 102 mg of phenolics for a human of 60 kg body weight. This dose equates to a 50% of cocoa polyphenols required to lower systolic blood pressure by about 5 mmHg and diastolic by about 3 mmHg as reported in clinical studies [42].

Taking into account that chronic administration of flavonoids such as quercetin has been shown to reduce blood pressure and enhance endothelium-dependent relaxation in various animal models of hypertension [35], it is highly possible that quercetin glycosides could be involved in the antihypertensive effect of the lyophilized BCAE. Moreover, in agreement with earlier published studies that point out that quercetin did not modify blood pressure in control normotensive animals, our results indicated that normotensive rats had no change on SBP with the treatment [13].

In addition, different phenolic compounds such as chlorogenic acid, anthocyanins, proanthocyanidins, and other flavonoids have been reported to have antihypertensive properties [35]. Therefore, the antihypertensive effect induced by the aqueous black cherry fruit extract could be a synergistic effect of chlorogenic acid, cyanidin-3-O-rutinoside, proanthocyanidins, and quercetine glycosides identified in this extract.

**Figure 6:**
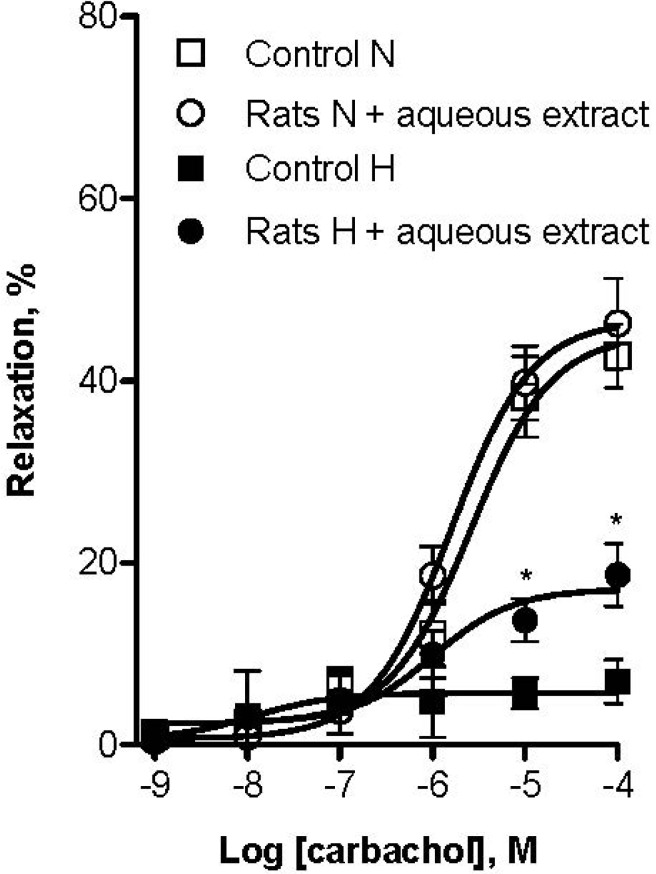
Effect of long-term oral administration of aqueous extract of black cherry fruits (300 mg/Kg/day) on vascular reactivity after four weeks treatment. Concentration-response curves are presented as percentage of relaxation of rat aortic rings pre-contracted by L-phenylephrine (1 µM) as a function of carbachol (CCh) concentration. L-NAME hypertensive induced rats group that received black cherry extract showed better response to CCh than L-NAME hypertensive induced rats group that not received black cherry extract. Normotensive rats groups had no significant difference from each other in their vascular reactivity. Each individual value is a mean ± SEM (*n* = 6). * *p* ˂ 0.05 *vs*. control.

Endothelial dysfunction has been associated with most forms of cardiovascular diseases such as hypertension [43]. Thus, we evaluated the improvement of endothelial function in L-NAME hypertensive rats following administration of aqueous black cherry fruit extract. After treatment, vascular reactivity of aortic rings was examined for each group of rats. The CCh vasodilator response on aortic rings precontracted with 1 µM Phe was used as an indicator of endothelial function. As shown in Figure 6, the relaxant effect of CCh was strongly diminished in L-NAME induced hypertensive rats compared to normotensive rats (E_max_ = 6.9% ± 1.6% and E_max_ = 42.6% ± 2.9%, respectively). However, the animals that received aqueous extract of black cherry fruits presented a better CCh relaxant response (E_max_ = 18.6% ± 1.7%). In contrast, the relaxant effect of CCh had no change in normotensive rats that received the aqueous extract compared to the control group (E_max_ = 46.2% ± 2.5%). This indicates that altered endothelial function in hypertensive animals was improved with this treatment. Taking into consideration that endothelial dysfunction has been associated with lower nitric oxide generation and oxidative excess [43], improvement of endothelial function could be due to the antioxidant capacity and the vasodilator effect induced by the black cherry fruit extract. To our knowledge, this is the first time that black cherry fruit has been studied for its vasodilator and antihypertensive properties.

## 3. Experimental

Reagents and standards were purchased from Sigma-Aldrich (St. Louis, MO, USA) unless stated otherwise. Methanol, acetonitrile (both HPLC grade), and formic acid (99% purity reagent grade) were obtained from JT Baker (Baker Mallinckrodt, Mexico City, Mexico).

### 3.1. Black Cherry Samples

Black cherry fruits were obtained from orchards in Huejotzingo, Puebla (México) in May 2011, they were transported to Querétaro, Qro., and stored at −70 °C. Voucher specimens (*P. serotina* voucher no. 825), identified by Dr. M. Martínez and Dr. L. Hernández-Sandoval, have been deposited in the Ethnobotanical Collection of the Herbarium of Querétaro “Dr. Jerzy Rzedowski”, located at the Faculty of Natural Sciences, University of Querétaro, Mexico. The whole fruits were lyophilized and some fruits were peeled to separate flesh and peel and both were also lyophilized separately.

### 3.2. Plum and Grape (Red Seedless) Samples

Fruits were obtained from a local market in Querétaro (México) and they were lyophilized.

### 3.3. Proximate Analysis and Mineral Content

Proximate composition of black cherry fruits, plums and grapes were analyzed by using AOAC methods for moisture (925.10), crude protein (960.52), crude fat (920.39), ash (900.02), and dietary fiber (985.29). Carbohydrates were determined according to the difference method by subtracting the other components from 100. All analysis were performed in triplicate and values averaged. Ca, Na, K, and Mg content of black cherry fruits, plums and grapes was determined by the dry-ash method 968.08 (AOAC). Phosphorous was determined according to method 965.17 (AOAC).

### 3.4. Extraction and Quantification of Total Phenolic Content

Total soluble phenolic compounds were extracted from the peel, flesh, whole black cherry fruit, plum, and grape as reported by Rivera-Pastrana *et al*. [44], using 20 mL of an extraction solution containing 80% methanol and 2% formic acid (v/v). Total phenolic content was analyzed by the Folin-Ciocalteu method [45]. Results were expressed as mg gallic acid equivalents (GAE)/100 g fresh weight (FW). Flavonoid content, expressed as mg catechin equivalents (CA)/100 g FW, was determined as reported by Cantín *et al*. [46].

### 3.5. Determination of Antioxidant Capacity

The antioxidant capacity (AOC) was measured by the 1,1-diphenyl-2-picrylhydrazyl (DPPH) radical scavenging method and ferric reducing ability of plasma (FRAP) assay as reported by Corral-Aguayo *et al*. [47]. AOC obtained from both assays was expressed as µmol Trolox equivalents (TE)/100 g FW.

### 3.6. HPLC/DAD/ESI-MS Analysis

This analysis was performed using a HP1100 series HPLC system (Hewlett-Packard GmbH, Waldbronn, Germany) coupled to a HP6210 time-of-flight (TOF) mass spectrometer (MS) (Agilent, Palo Alto, CA, USA) [44]. Chlorogenic acid content was obtained using a calibration curve of pure standard (0.001–0.1 g L^−1^) and quercetin glycoside content were obtained based on a quercetin standard calibration curve (0.0002–0.0012 g L^−1^).

### 3.7. Preparation of Aqueous Extracts

The aqueous extracts were prepared by adding to whole pitted fruits, peel, and flesh of black cherry hot distilled water (1:10 w/v) over 20 min. The hot water extracts were filtered through number 1 Whatman paper. The whole black cherry aqueous extract (BCAE), black cherry peel aqueous extract (BCP), and black cherry flesh aqueous extract (BCF) were lyophilized and stored at −20 °C for later use in *in vitro* and *in vivo* evaluations.

### 3.8. Determination of the Vasorelaxant Effect

The vasorelaxant effect was measured on the isolated rat aortic rings assay as described by Ibarra-Alvarado *et al.* [22]. Acetylcholine (ACh) and the lyophilized black cherry aqueous extracts (BCAE, BCP, and BCF) dissolved in deionized water were added to the organ bath at final concentrations of 0.1–1,000 µg/mL and 1–1,000 µg/mL, respectively, to obtain concentration-response curves. The isometric tension was measured by a Grass FT03 force-displacement transducer attached to a Grass 7D polygraph.

### 3.9. Determination of the Antihypertensive Effect

All animal experiments were performed in accordance with Federal Regulations for Animal Experimentation and Care (Mexican Official Standard NOM-062-ZOO-1999).

#### 3.9.1. Experimental Schedule and Blood Pressure Measurements

Hypertension was induced in rats by oral administration of Nω-nitro-l-arginine methyl ester (L-NAME) during four weeks according to Belmokhtar *et al*. [48]. Adult male Wistar rats (250–300 g) were divided into four groups (*n* = 6): the control group received tap water; the second group received tap water with lyophilized BCAE (300 mg/kg body weight/day); the third one received tap water with L-NAME (30 mg/kg body weight/day); and the last one received tap water with L-NAME (30 mg/kg body weight/day) plus lyophilized BCAE (300 mg/kg body weight/day). The systolic blood pressure (SBP) was measured weekly using the tail-cuff method with a LE 5650/6 heater scanner (Panlab, Barcelona, Spain) coupled to LE 5007 automatic blood pressure computer (Letica, Barcelona, Spain). The mean value from at least five consecutive readings was used for the calculations.

#### 3.9.2. Effect of Long-Term Oral Administration of the Lyophilized Aqueous Extract (BCAE) on Vascular Reactivity: *Ex Vivo* Experiments

At the end of each treatment, thoracic aortas from all groups were isolated to determine the effect of different treatments on vascular reactivity by means of the isolated rat aorta assay [48]. After equilibration, aortic rings were contracted with 1 µM phenylephrine (PE). When the steady contraction was reached, the response to increasing concentrations of carbachol (CCh) (10^−9^–10^−4^ M) was tested. Relaxation was expressed as percent of the maximal PE-induced contraction.

### 3.10. Statistical Analysis

#### 3.10.1. Proximate Analysis, Phenolic Content and Antioxidant Capacity

Results of the experiments are expressed as the mean ± standard deviation (SD) from *n* = 3 − 9 experiments. The data were analyzed by a one-way ANOVA and the Tukey test. Differences between the means were considered to be significant when *p* ˂ 0.05. Pearson’s correlation coefficient (*r*) was calculated in order to measure the association between phenolic content and antioxidant capacity.

#### 3.10.2. *In Vitro* Assays

Results of the experiments are expressed as the mean ± standard error of the mean (SEM) from *n* = 6 experiments. Concentration-response curves for the extracts were plotted and fitted to a sigmoidal equation.

#### 3.10.3. *In Vivo* Assays

The results are expressed as mean values ± SEM from *n* = 6 experiments. To compare the different treatments, data were analyzed by a one-way ANOVA, and differences between the groups were assessed by the Tukey test. Differences between the means were considered to be significant when *p* < 0.05. The data analysis and graphics were achieved with the program Prism 4.0 (GraphPad Software, San Diego, CA, USA).

## 4. Conclusions

These results suggest that black cherry fruit has a good antioxidant capacity, which could be accounted for its polyphenol content. Additionally, this fruit contains compounds such as hyperoside and chlorogenic acid that elicit antioxidant, vasodilator and antihypertensive effects. Equally important are the results from proximate and mineral analyses, which showed that black cherry fruit has high protein and mineral contents. For these reasons, black cherry fruit may be used as a functional food, which could be potentially useful in the prevention and treatment of hypertension.
